# Gut Microbial Flora, Prebiotics, and Probiotics in IBD: Their Current Usage and Utility

**DOI:** 10.1155/2013/435268

**Published:** 2013-08-07

**Authors:** Franco Scaldaferri, Viviana Gerardi, Loris Riccardo Lopetuso, Fabio Del Zompo, Francesca Mangiola, Ivo Boškoski, Giovanni Bruno, Valentina Petito, Lucrezia Laterza, Giovanni Cammarota, Eleonora Gaetani, Alessandro Sgambato, Antonio Gasbarrini

**Affiliations:** ^1^Department of Internal Medicine, Gastroenterology Division, Catholic University of Sacred Hearth, Policlinico “A. Gemelli” Hospital, lgo Gemelli 8, 00168 Roma, Italy; ^2^Institute of Pathology, Catholic University of Sacred Hearth, lgo Gemelli 8, 00168 Rome, Italy

## Abstract

Inflammatory bowel diseases are chronic diseases affecting the gastrointestinal tract, whose major forms are represented by Crohn's disease (CD) and ulcerative colitis (UC). Their etiology is still unclear, although several factors have been identified as major determinants for induction or relapses. Among these, the role of the “forgotten organ”, gut microbiota, has become more appreciated in recent years. The delicate symbiotic relationship between the gut microbiota and the host appears to be lost in IBD. In this perspective, several studies have been conducted to assess the role of prebiotics and probiotics in gut microbiota modulation. This is a minireview aimed to address in an easy format (simple questions-simple answers) some common issues about the theme. An update on the role of selected constituents of gut microbiota in the pathogenesis of IBD is presented together with the analysis of the efficacy of gut microbiota modulation by prebiotics and probiotics administration in the management of IBD.

## 1. Introduction

The human microflora, known as “microbiota”, includes bacteria, fungi, bacteriophages, and viruses and acts as an “organ” synergistically with the host, creating an ecosystem. It is able to colonize skin, the genitourinary system, the respiratory system, and, above all, the gut.

Gut microbiota includes around a thousand different species and more than 15,000 different strains of bacteria, for a total weight of about 1 Kg. Stomach and small intestine are relatively poor of bacteria, whilst the colon hosts about 10^12^ microorganisms [1], mainly belonging to the Firmicutes and Bacteroidetes phyla [2]. Other domains represented are those of Archaea [3] and Eukarya, plus many viruses and bacteriophages [4]. Gut microbiota is harbored also by several yeast families, whose role in gastrointestinal physiology as well as in diseases still remains unclear.

Since most species seem to be refractory to cultivation with usual methods, culture-independent molecular techniques, such as 16S rDNA genotyping, are used to characterize the gut microflora from both fecal samples and bowel biopsies [5–8].

At birth, the human gut is sterile, and the first colonization occurs during childbirth and the first feed. Subsequently, the microbiota changes under the influence of age, sex, state of immune maturation, and environmental factors. After the first two years, the microbiota becomes more stable, although a stable endogen flora could be differentiated by a transient one, which is, on the contrary, more sensitive to external stimuli, as the gut mucosa is the first line of communication with exogenous agents [9, 10].

During the first years of life, gut microbiota stimulates the innate immunity, by inducing gut-associated lymphoid system, and the acquired immunity, by stimulating specific systemic and local immune responses [11]. In the gut, bacterial fragments stimulate specific receptors, like TLR9 (toll-like receptor 9, expressed on epithelial and immune cells) and the inflammasome that are able to recognize bacterial DNA [12].

In normal conditions, stimulation of the mucosal immune system by gut microbiota determines a state of “low-grade physiological inflammation” [13], a status of continues activation of the mucosal immune system in response to commensals, and, in case of needs, also towards pathogens. Mucosal homeostasis requires a continuous balance between pro- and anti-inflammatory components. In recent years several studies investigated the correlation between dysbiosis and intestinal and extraintestinal diseases, including immune system alteration, obesity, allergies, autoimmune diseases, irritable bowel syndrome (IBS), and inflammatory bowel disease (IBD) [14, 15].

IBD are chronic, relapsing, multifactorial conditions affecting the digestive tract. These majorly include ulcerative colitis (UC) and Crohn's disease (CD). Although the etiology of these diseases is still unclear, the main hypothesis is that IBD are a result of an excessive immune response to endogenous bacteria, which occurs in genetically predisposed individuals [16, 17]. 

Most of conventional IBD therapies aim to modulate immune system. 5-aminosalicylic acid (ASA) compounds, corticosteroids, azathioprine/6-mercaptopurine, methotrexate, cyclosporine, and anti-TNF*α* agents are constantly used to manage these diseases. Several and probably less characterized therapies, as additive to or alternative to conventional therapies, in milder cases, aim to modulate gut microbiota, directly or indirectly. For example, antibiotics are used in IBD, and they are considered particularly effective in perianal and postoperative CD and in pouchitis [18]. 

Probiotics contain viable organisms, sufficient amounts of which reach the intestine in an active state, thus exert positive health effects [19]. They mostly include lactic acid-producing bacteria and yeasts that reach the gut unaltered, without providing damage to the host [18, 20]. Their mechanisms of action are still unclear; they probably modulate the membrane permeability and the mucosal immune system, keeping away pathogens from intestinal mucosa surface. *Lactobacillus* and *Bifidobacteria* produce harmful substances for Gram-positive and Gram-negative bacteria, and they compete with pathogens (i.e., *Bacteriodetes, Clostridium, Staphylococcus,* and *Enterobacter*) for cell adhesion [18, 21, 22].

On the other side, prebiotics are selectively fermented ingredients that allow specific changes both in the composition and/or in the activity of gastrointestinal microflora, conferring benefits upon the host well-being and health [19]. They are nondigestible oligosaccharides, such as fructooligosaccharides (FOS), galactooligosaccharides (GOS), lactulose, and inulin, and they have the potential to stimulate growth of selective and beneficial gut bacteria [18]. Because of their composition, they cannot be adsorbed until they reach colon, where they can be fermented by a specific microflora into small chain fatty acid (SCFA) and lactate [23]. 

**Figure 1:**
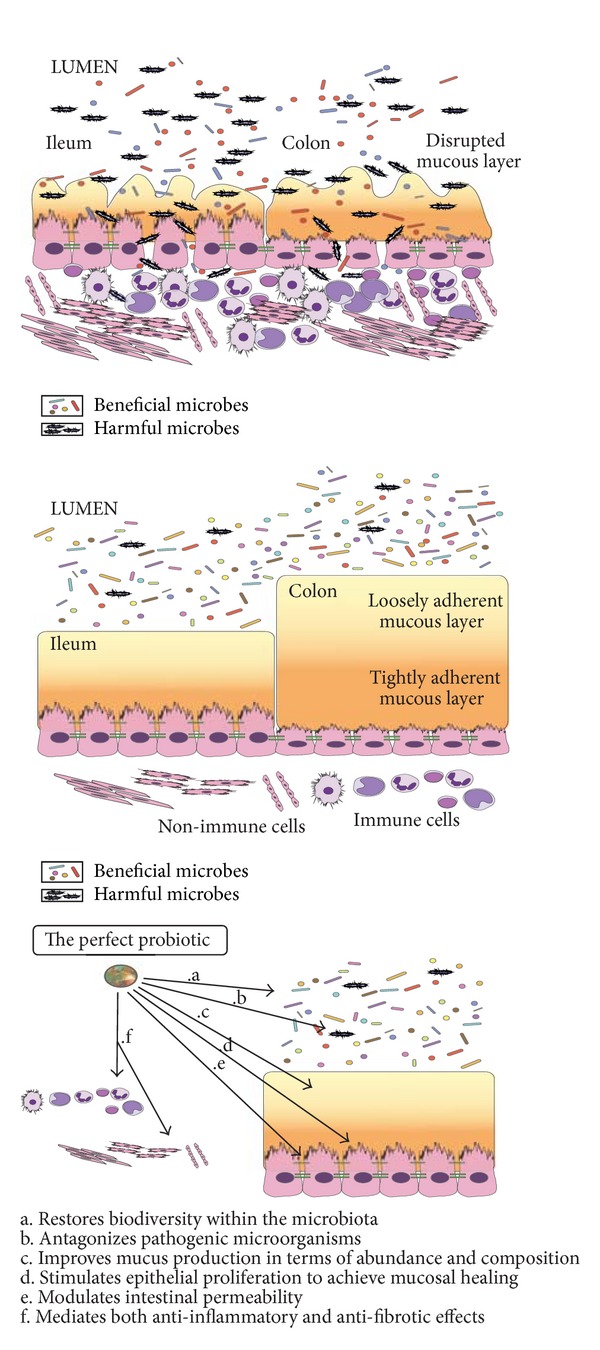
Gut microbiota in health condition and IBD and functions of “the perfect probiotic”.

Their exact mechanism of action is still unclear. Recent evidences hypothesized that they are able to increase the production of SCFA and to modulate cytokines production within the gut mucosa, by modulating the gut flora composition. The synergistic combination of pro- and prebiotics is called “synbiotic” [19] (Figure 1).

## 2. Is There a Role for Specific Pathogens in IBD?

It was originally suspected that IBD depend on a single pathogenic strain of bacteria. In 1984, Chiodini et al. [24] showed a strong association between *Mycobacterium avium subspecies paratubercalosis* (MAP) and CD, but this hypothesis was confirmed only by few studies. Furthermore, the inefficiency of antituberculosis antibiotics in CD patients reinforced the criticism towards MAP [25, 26].

*Escherichia coli* is usually isolated in many intestinal biopsies of CD patients. In particular, adherent-invasive strains (AIEC) are found in patients with ileal CD [27]. Also *Yersinia* and *Pseudomonas* are supposed to act as triggers in CD disease [28]. On the other side, *Salmonella, Campylobacter jejuni, Clostridium difficile, Adenovirus,* and *Mycoplasma* have been identified as agents associated to disease relapsing but not to induction [29, 30]. 

A study conducted by Willing et al. [31] showed that, in ileal CD patients, *Faecalibacterium *and *Roseburia* are underrepresented whilst *Enterobacteriaceae* (such as *E. coli*) and *Ruminococcus gnavus *are increased.

*Fusobacterium varium* has been localized in the colon of UC patients and causes UC in mice when injected by enema [32, 33]. Moreover, it has been assessed an overgrowth of *E. coli* in UC patients, suggesting a possible role on genesis and/or maintenance of the disease [34].

### 2.1. Is There a Role of the Commensal Flora in IBD Pathogenesis?

Several evidences support the hypothesis that gut microbiota plays a role in the pathogenesis of IBD, particularly studies involving animal models or in vitro models. Here we decided to present limited data coming from experimental models, while focusing more on human studies. 

The most inflamed intestinal areas in IBD patients are the same displaying the highest amount of intestinal bacteria. The evidence that germ-free mice do not develop severe colitis supports this finding [35]. Furthermore, recurrence rate of postoperative CD and pouchitis is higher when the fecal stream is reestablished [15, 36]. 

IBD patients display a reduced amount of dominant commensal bacteria, such as *Firmicutes* (in particular *Clostridium clusters IX and IV*) and *Bacteriodetes*, facing an increased number of *Proteobacteria* and *Actinobacteria*. This observation is associated with a decreased SCFA level in feces of IBD patients. Among SCFAs, a decrease in butyrate level has been associated with IBD as it is able to inhibit proinflammatory cytokines release to increase the production of mucin and antimicrobial peptides and to provide energy to colonocytes [37–39].

Among *Firmicutes*, the reduction of *Faecalibacterium prausnitzii* has recently emerged as a very frequent finding in CD; as shown in Sokol et al., administration of *Faecalibacterium prausnitzii* has an anti-inflammatory activity, as demonstrated by in vivo and in vitro studies [37]. 

Similar findings were obtained by Joossens et al. Fecal microbiota of 68 CD patients, 84 unaffected relatives, and 55 controls were analyzed, and dysbiosis in CD patients was found. In particular, it was assessed a reduction of *Faecalibacterium prausnitzii*, *Bifidobacterium adolescentis*, *Dialister invisus*, and of an unknown species of *Clostridium clusters XIVA*, while *Ruminococcus gnavus* was increased. This report is the largest population study focused on gut microbiota composition in IBD, where relatives represented controls and differences were detectable despite common habits and genetics. Moreover, a different microbiota composition was assessed in IBD relatives compared to controls because of the higher prevalence of bacteria with mucin degradation capacity [40].

A different gut microbiota was found also in patients with pouchitis compared to controls with a decreased concentration of *Bacteriodetes* and *Faecalibacterium prausnitzii* and an increase in *Proteobacteria* [41, 42].

Furthermore, higher level of sulphate-reducing bacteria (SRB) has also been observed in IBD, mainly in UC and pouchitis patients. SRB are associated to a higher hydrogen sulphide level, but to a less butyrate production. This is supposed to induce cell hyperproliferation [43]. Interestingly, SRB are supposed to be crucially important to induce DSS colitis in mice [44]. Among these, a study conducted by Rowan et al. [45] demonstrated an increased number of *Desulfovibrio* subspecies in acute and chronic ulcerative colitis with their products inhibited by the use of 5-aminosalycilic acid [46].

The influence of microbiota in IBD is also supported by the potential role of fecal transplantation, efficiently utilized in severe *Clostridium difficile* infection, but also in IBD, particularly in UC patients [47, 48].

On the other side, the importance of fungal flora in IBD is still unclear. The presence of *Saccharomyces Cerevisiae* antibodies in CD patients offered a starting point for reflection on the role of fungi in IBD pathogenesis. Enlarged populations of *Candida spp.*, *Penicillium spp.,* and *Saccharomyces sp.* were found in IBD patients compared to controls. However, more studies are necessary to investigate whether fungal diversity in IBD is a trigger for disease initiation or rather a secondary effect of changes in bacterial composition and therapy [49] (Table 1). 

## 3. Does Appendectomy Affect the Clinical Course of IBD?

A major role in the pathogenesis of IBD seems to be related to the functions of cecal appendix, not to be considered as a vestige [51], but rather one of the most important immune organs along the gastrointestinal tract, as firstly suggested by Berry [52]. Appendix maintains the homeostasis of gut microbial flora by producing and shedding biofilms with the aim of modulating the epithelial regeneration and protecting from pathogen microbes [53–55]. The big amount of appendicular lymphoid tissue determines the introduction into the cecal lumen of compounds such as mucin and IgA. The evidence of an increased concentration of SIgA in fecal samples of IBD patients—especially in CD patients—toward healthy controls [56] confirms the central role of the immune system in the pathogenesis of these inflammatory diseases. Several studies in literature try to solve the issue of establishing the weight of appendix and appendectomy in IBD. The majority of them support a highly significant inverse relationship between appendectomy and the need for surgery and immunosuppressant in UC patients [57–59], with no significant variation in activity outcomes [60]. To date, few studies about the relationship between appendix, appendectomy, and CD show controversial results [61].

## 4. Which Role for Pre- and Probiotics on the modulation of GUT Microbiota Composition?

Gut microbiota modulation can be obtained with several approaches, including antibiotics, pro- and prebiotics supplementations, diet and correction of predisposing factors responsible for gut microbiota alterations. Despite the simplicity of this statement, few reports are really addressing the ability of these factors to modulate gut microbiota composition. 

Venturi et al. reported that use of VSL#3 on 20 UC patients intolerant or allergic with 5-ASA was associated with an increase in fecal concentrations of *Streptococcus salivarius ssp. thermophilus*, *Lactobacilli,* and *Bifidobacteria*, which remained stable throughout the study. After 15 days from discontinuation, levels returned similar to the basal ones. Conversely, no change in fecal concentration of *Bacteroides, Clostridia*, coliforms, aerobic, and anaerobic bacteria was reported [62]. Cui et al. demonstrated that treatment with BIFICO induced an increase in *Bacilli*,* Enterococci*, *Bifidobacteria,* and *Lactobacilli*, with a decrease of *Bacteroides* and *Bifidobacteria* [63].

Finally, Welters et al. demonstrated a reduction in the number of *Bacteriodetes* in feces of patients with chronic pouchitis treated with 24 g per day of inulin [64].

The majority of the lines of evidence on the ability of probiotics and prebiotics in modulating gut microbiota come from indirect studies showing clinical efficacy of those in IBD, which will be synthetically reported below.

## 5. Any Issues Related to the Methodology Used for Studying Gut Bacteria?

Gut bacteria do not grow in regular culture media. That was probably associated in the past with a clear underestimate of gut microbiota role in human health and disease New techniques involving culture-independent molecular techniques, mostly related on analysis of 16S rDNA and including RT-PCR, pyrosequencing, or microarray opened new horizons in this field [5–8]. The abundance of gut microbiota in human body suggests that we are mostly made of bacteria [2], and in the future we will probably realize that we are an image of the balance between ourselves and bacteria within us.

## 6. Can the Modulation of Gut Microbiota Cure IBD?

### 6.1. Probiotics and IBD

There are few studies on the efficacy of probiotics in CD. These studies include a small number of patients. Only one study is included in a Cochrane review of randomized controlled trials. It compared the efficacy of *Lactobacillus GG* toward placebo in CD patients. Eleven patients were selected, only 5/11 completed the study, and no significant differences were observed between the two groups [65]. In 2005, Bousvaros et al. conducted a randomized double-blind placebo controlled trial to establish the efficacy of *Lactobacillus GG* as adjuvant to standard therapy in the maintenance of remission in seventy-five CD patients. No difference between the two groups was found [66]. Moreover, *Saccharomyces boulardii* showed positive effects on maintaining a longer remission in CD [77] and improving the intestinal barrier permeability [78]. Afterwards, a Cochrane review, on seven studies [67], and a recent meta-analysis [68], on eight randomized placebo-controlled clinical trial, confirmed that probiotics are useless in maintaining remission and preventing recurrence in CD. Their inefficiency is tested also for postoperative CD [69].

For UC, a different scenario is described. The efficacy of VSL#3 (*Bifidobacterium breve, Bifidobacterium longum, Bifidobacterium infantis, Lactobacillus acidophilus, Lactobacillus plantarum, Lactobacillus paracasei, Lactobacillus bulgaricus, and Streptococcus thermophilus*) in UC patients was proved in several papers. Bibiloni et al. studied 34 adult patients with mild-moderate UC, in absence of adverse events [71]. Venturi et al. assessed its positive effect on 20 UC patients intolerant or allergic to 5-ASA [62]. VSL#3 efficacy was also tested in a study conducted on children with newly diagnosed UC [72]. In 2004, Kruis et al. showed that there was no difference in the use of the probiotic *E. coli Nissle 1917* and mesalazine in the maintenance of remission in UC patients. On the other side, Cui et al. demonstrated the efficacy of BIFICO in preventing flares in UC patients [63, 73]. In 2006, another study showed the efficacy of *Lactobacillus GG* in maintenance of remission in 187 UC patients [70]. In 2007, in a Cochrane review, Mallon et al. [79] concluded that probiotics could provide efficacy in the maintenance of remission in patients with mild-moderate UC, while limited efficacy could be predicted for moderate-severe disease. Despite these positive results about the efficacy of probiotics in the maintenance of remission in UC, further studies are necessary [74, 80].

In pouchitis, Gionchetti et al. sustained VSL#3 efficacy in a study conducted on 40 patients with ileal pouch-anal anastomosis. Twenty patients received VSL#3 and 20 received placebo: only 10% of patients that received VSL#3 developed pouchitis versus 40% of placebo patients [75]. Mimura et al. confirmed these data [76].

Based on this, VSL#3 is approved for the prevention and the maintenance of remission of pouchitis, and the efficacy is stated also in referral European guidelines [18, 74] (Table 2).

### 6.2. Prebiotics and IBD

The efficacy of prebiotics in IBD is mostly confined to in vitro [87] and animal models (DSS and TNBS-induced colitis) studies [88–91]. However, there are also few human studies that include a small number of patients.

One of the first studies conducted involved 10 CD patients receiving 15 g of fructooligosaccharides (FOS). In these patients, the disease activity index was reduced, and mucosal *Bifidobacteria* were increased [81]. The efficacy of FOS in CD was also tested by Benjamin et al. in a study published in 2011. On hundred and three CD patients were randomized to receive 15 g/day FOS or placebo for 4 weeks. There was no significant clinical improvement in patients receiving FOS, but they had reduced proportions of interleukin (IL)-6-positive lamina propria dendritic cells (DC) and increased DC IL-10 staining. There was no change in IL-12p40 production. Significant difference in the number of *Bifidobacteria* and *F. prausnitzii* in feces was not observed [82].

In 2002, Bamba et al. demonstrated a potential role of germinated barley foodstuff (GBF) in inducing remission in patients with mild to moderate active ulcerative colitis [83]. The same result was confirmed by a study conducted by Kanauchi et al. [84]. The potential efficacy of another prebiotic, *Ispaghula husk*, was found by Hallert et al. [85]. A prospective, randomized, placebo controlled pilot trial on 19 UC patients treated with mesalazine showed that the group who received oligofructose-enriched inulin supplementation had a lower fecal calprotectin than controls. Fecal calprotectin is an inflammatory marker, so we can suppose that prebiotics can reduce inflammation in UC patients [86].

Furthermore, Welters et al. demonstrated that inulin supplementation in pouchitis was associated to a lower inflammation indicated by an increased level of butyrate, a lower concentration of *Bacteroides fragilis* and secondary bile acids in feces, and a reduced endoscopic inflammation [64] (Table 3). 

## 7. Limitations and Future Perspectives

Nevertheless, several doubts and limitations remain unsolved. Gut microbiota composition in healthy individuals is still unclear. There are no studies with a primary aim focused on a specific therapy towards the modifications of gut microbiota. Probiotics use is often not evidence based because mechanisms of action are still unclear, such as intestinal bioavailability of bacterial strains, dose, and treatment time. Moreover, useful methods for gut microbiota characterization have high cost and are not standardized. Future practice will probably provide a gut microbiota characterization, which will be useful for different types of application. Pre- and/or probiotic therapy will consist in supplementation of specific subset of bacterial strains, which will provide the desired variation in gut microbiota composition.

## 8. Conclusions

Gut microbiota plays a crucial role in triggering, maintaining, and exacerbating IBD. Specific microbes can be overrepresented in IBD while others seem to be protective. A decrease in microbial biodiversity has been found in mucosa and feces of IBD patients, together with an increase of fungi.

Pre- and probiotics could represent a valid armamentarium to modulate gut microbiota and, probably, to cure IBD. Current evidences, however, show a clear clinical efficacy of some families of probiotics only in pouchitis and ulcerative colitis but not in Crohn's disease. This efficacy has been prevalently associated to mild disease and seems to have a better role in maintenance of remission compared to induction of remission.

Further studies are necessary to better characterize the exact role of probiotics in IBD, their specific mechanisms of actions, including a direct effect on mucosal homeostasis or healing. Since probiotics are becoming a legitimate therapeutic option, it is necessary to determine which probiotic strains have the greatest efficacy, whether they are more effective alone, or in conjunction with other pro- or prebiotics, and what is their half-life in the gastrointestinal tract. On the base of these data, frequency of administration and dose could be exactly calculated. 

## Figures and Tables

**Table 1:** Dysbiosis and IBD.

Bacteria	CD	UC	References
*Escherichia coli *	↑	↑	[27, 31, 34]
*Pseudomonas *	↑		[50]
*Salmonella *	↑ (relapsed)		[29, 30]
*Campylobacter jejuni *	↑ (relapsed)		[29, 30]
*Clostridium difficile *	↑ (relapsed)	↑	[29, 30, 47, 48]
*Fusobacterium varium *		↑	[32, 33, 37]
*Clostridium *cluster IX	↓	↓	[37]
*Clostridium *cluster IV	↓	↓	[37]
*Faecalibacterium prausnitzii *	↓		[31, 37, 40–42]
*Bifidobacterium adolescentis *	↓		[40]
*Dialister invisus *	↓		[40]
*Ruminococcus gnavus *	↑		[40]
*Enterobacteriaceae *	↑	↑	[27, 31, 34]
*Firmicutes *	↓	↓	[37, 39, 41, 42]
*Bacteroidetes *	↓	↓	[37, 39, 41, 42]
*Proteobacteria *	↑	↑	[37, 39, 41, 42]
*Actinobacteria *	↑	↑	[37]
*Sulphate-reducing bacteria *(SRB)		↓	[43, 44, 46]

**Table 2:** Probiotics and IBD.

Probiotics and IBD	Aim	Conclusion	References
*Lactobacillus GG *	Adjuvant to standard therapy in maintenance of remission in CD	Useless	[65–69]
	Adjuvant to standard therapy in maintenance of remission in UC	Effective	[70]
VSL#3	Adjuvant to standard therapy in maintenance of remission in UC	Effective	[63, 71–73]
	Prevention of pouchitis	Effective	[18, 74–76]
*Saccharomyces boulardii *	Adjuvant to standard therapy in maintenance of remission in CD	Effective	[77]

**Table 3:** Prebiotics and IBD.

Prebiotics and IBD	Aim	Conclusion	References
15 g fructooligosaccharides (FOS)	Reduction of disease activity index	Controversial data	[81, 82]
Germinated barley foodstuff (GBF)	Remission in patients with mild-to-moderate active UC	Effective	[83, 84]
*Ispaghula husk *	Remission in patients with mild-to-moderate active UC	Effective	[85]
Oligofructose with inulin	Reduction of inflammation in UC	Effective	[86]
Inulin	Reduction of inflammation in pouchitis	Effective	[64]
